# Dupuytren’s Disease Percutaneous Needle Aponeurotomy: Does Grip Strength Improve Post Procedure?

**DOI:** 10.3390/jcm14124171

**Published:** 2025-06-12

**Authors:** Jessica Medland, Nicole Garcia, Ishith Seth, Warren M. Rozen

**Affiliations:** 1 Department of Plastic and Reconstructive Surgery, Frankston Hospital, Peninsula Health, Frankston, VIC 3199, Australiawarrenrozen@hotmail.com (W.M.R.); 2 Peninsula Clinical School, Faculty of Medicine, Monash University, Frankston, VIC 3800, Australia

**Keywords:** Dupuytren’s disease, percutaneous needle aponeurotomy, grip strength, hand function, dynamometer

## Abstract

**Background/Objectives:** Dupuytren’s disease (DD) presents significant challenges in hand function due to the progressive contracture of the palmar fascia. This study evaluates the impact of Percutaneous Needle Aponeurotomy (PNA) on grip strength before and after intervention. **Methods**: A prospective pilot study included patients with DD over 18 years of age who underwent PNA. Grip strength was measured at baseline, six weeks, and three months post-procedure using a Jamar Dynamometer. The median time to return to work or normal activities was recorded. **Results:** The study included a total of 29 participants. There was a recorded difference in one kilogram of baseline grip strength between the treatment and non-treatment hands. Patients reported improved hand function and recorded an average increase of 5.8 kg in grip strength at the three-month follow-up. Improvements were demonstrated in active extension, averaging 26 degrees at the metacarpophalangeal joint and 27 degrees at the proximal interphalangeal joint. Nine minor skin tears occurred, and there was no recorded recurrence. **Conclusions**: This study adds to the literature, confirming PNA is a safe and effective intervention for DD, offering rapid recovery and functional improvement. A larger study of a longer duration will help to establish whether grip strength gains are maintained following PNA.

## 1. Introduction

Dupuytren’s disease (DD) is a chronic condition characterised by the pathological thickening and contraction of the palmar fascia. The disease can significantly impact a patient’s hand function, including grip strength, thereby affecting activities of daily living, social interactions, and quality of life [1]. Bulky nodules or thick palmar cords can progress to flexion deformities with associated stiffness that impacts an individual’s grip strength and in-hand manipulation of objects. Ultimately, this results in patient frustration, decreased work productivity, and lowered self-esteem [2]. Given these challenges, treatments for DD aim not only to improve finger extension and hand appearance but also to enhance functional outcomes, including grip strength.

Percutaneous Needle Aponeurotomy (PNA) is a minimally invasive and ideal approach in this context. PNA involves the percutaneous division of the fibrous cords that cause finger contractures, utilising a needle’s edge [3]. This technique has historical roots dating back to the 18th century and has been refined over the years to provide a simple, effective, and swift intervention for DD. PNA’s efficacy, coupled with a lower risk of complications and the ability to treat multiple digits and joint contractures in a single session, makes it an attractive option for patients seeking management of their DD [4]. Furthermore, PNA offers the potential for rapid recovery and minimal downtime, significant advantages over more invasive surgical options.

Beyond aesthetic and range-of-motion improvements, one of the most significant measures of success in treating DD is the restoration of hand function, particularly grip strength and dexterity. Grip strength is crucial for performing daily tasks, such as opening jars, using tools, and carrying bags. In DD patients, contractures in the metacarpophalangeal joint (MCPJ) and proximal interphalangeal joint (PIPJ) diminish both the force and the coordination needed for effective hand use. By releasing these contractures, PNA may lead to measurable improvements in grip strength, which directly translates into greater functional independence and quality of life.

Previous studies have suggested that patients often report a subjective improvement in hand function following PNA [5], while objective assessments tend to focus on extension deficits rather than grip strength [2,6,7,8,9,10,11]. Functional gains are not only immediate but may also be sustained over time, especially when combined with post-procedure rehabilitation or hand therapy. Importantly, these improvements occur with relatively low morbidity, reinforcing PNA’s role as a function-preserving intervention.

This study hypothesises that PNA enhances mobility, reduces flexion deformities, and diminishes the prominence of palmar nodules, ultimately improving grip strength and overall hand function. Through a prospective analysis, the research aims to investigate the impact of PNA on grip strength outcomes, offering deeper insights into its effectiveness in addressing the functional impairments associated with DD. While PNA is an established technique, few studies have directly measured grip strength before and after the procedure. This study provides prospective, quantitative data on grip strength improvement, offering functional validation of PNA beyond contracture correction.

## 2. Materials and Methods

A prospective study was conducted at a single institution to assess grip strength outcomes in patients with DD following PNA. An ethics committee reviewed and approved the study protocol (QA/106427/PH-2024-418696(v2)). All participants provided written informed consent before participating in the study. The research was conducted in accordance with the Declaration of Helsinki and the ethical guidelines for medical research involving human subjects.

Participants were selected from individuals undergoing PNA treatment for DD at the treating hospital. Eligibility criteria included adults aged 18 years and older diagnosed with DD and a Tubiana classification of grade I or higher, indicating the presence of a clinically measurable flexion contracture. The Tubiana classification is a validated system that stratifies DD severity based on the total passive extension deficit (PED) across affected joints. It ranges from Grade I (0–45°), representing mild disease, to Grade IV (>135°), indicating severe contracture. This study utilised Tubiana staging both as an inclusion criterion and as a tool to stratify disease severity, enabling a more structured evaluation of functional outcomes following PNA. Patients undergoing primary or repeat PNA procedures for DD were included in the study. Recurrence was not an exclusion criterion. Patients with a concurrent symptomatic hand condition affecting grip strength, such as trigger finger or carpal tunnel syndrome, on the treated hand were excluded. As a pilot study, no formal sample size calculation was performed. The sample size of 29 participants was determined based on available clinical volume and feasibility, to explore trends and inform future larger studies.

Demographic data collected at the baseline included age, gender, occupation, and hand dominance, with a particular focus on risk factors associated with DD, such as ethnicity, family history, and diabetes. Patients attended the outpatient clinic for a day procedure. PNA was performed by a single experienced hand surgeon following standard clinical protocols, with the patient awake and seated in a recliner chair. Palmar and digital cords were palpated and marked with a marking pen prior. Local anaesthetic injection of two percent lidocaine was administered via a 25-gauge needle to each affected digit around the palmar nodules and a standard ring block. The tip of a 21-gauge hypodermic needle was inserted close to parallel to the skin, bevel pointing distally, and the fibrous cords were broken in a levering motion upwards at several marked points, before the affected digit was manipulated into extension. The digit was held in extension by the clinician to maintain cord prominence, and meticulous care was taken to protect the neurovascular bundles. Patients without a diagnosis of diabetes mellitus were injected with 1 mL triamcinolone acetonide suspension 40 mg/1 ml post-procedure. The hand was gently cleaned, and a simple dressing of gauze and small crepe was applied to the affected digits. All patients attended hand therapy on the day of the procedure for exercises and the fabrication of a night extension splint that is worn for three months. Patients attended an average of two additional hand therapy sessions in the three-month follow-up period. Patients were scheduled for follow-up at six weeks and three months post-procedure. A minimum follow-up of three months was required for inclusion in the final analysis. The focus of therapy was to review the patient’s progress with maintaining extension and regaining flexion and their compliance with the night splinting regimen. There was no emphasis on prescribed strengthening exercises; instead, patients were encouraged to focus on using their hands in normal, functional tasks.

The primary outcome measure was the grip strength, which was assessed using a Jamar Dynamometer (Model 5030 J1, Sammons Preston Rolyan, Bolingbrook, IL, USA). Measurements were taken on both hands before the procedure to establish a baseline, then at six weeks and three months following PNA to evaluate changes over time. Grip strength was measured in kilograms-force (kgf) using a standardised protocol. The dynamometer was set at position two. Patients were seated with their shoulders adducted and neutrally rotated, elbows flexed at 90 degrees, forearms in a neutral position, and wrists between 0 and 30 degrees of extension. Each patient performed three trials with each hand, and the highest value was recorded. Secondary outcome measures included changes in active extension, assessed at baseline, six weeks, and three months using a goniometer. Negative values indicating hyperextension were corrected to zero. Information on hand dominance was collected to examine the impact of PNA on the dominant versus non-dominant hand. Finally, patient-reported outcome measures (PROMS), Southampton Unité Rhumatologique des Affections de la Main (URAM), were administered and timeframes for return to work or activity were documented. PNA complications were recorded to assess the safety and potential adverse effects of the procedure. 

Standard descriptive statistics summarised the study population’s demographic data and baseline characteristics. Changes in grip strength, contracture angles, and the incidence of complications were analysed using paired *t*-tests or Wilcoxon signed-rank tests, depending on the data distribution. A *p*-value of less than 0.05 was considered statistically significant. All statistical analyses were performed using STATA v19 or a similar statistical software package.

Although the inclusion criteria specified a minimum follow-up of three months, patients with partial follow-up (i.e., baseline and either six-week or three-month data) were retained in the analysis where applicable. This decision was made to preserve valuable outcome data and reflect the practical limitations of real-world follow-up in prospective clinical settings. For analyses involving three-month outcomes, only patients with complete data at that timepoint (*n* = 20) were included.

## 3. Results

Data was collected from February to July 2024. A total of 34 patients underwent a PNA procedure, including 56 individual digit contracture releases. Data for four patients were not included due to no record of baseline grip strength. One participant was excluded from the study due to proven carpal tunnel syndrome by nerve conduction tests. Of the remaining 29 participants, ages ranged from 46 to 92 years, including 24 males and 5 females. Baseline characteristics were recorded in Table 1.

In a single procedure, the number of digits released ranged from one to three. Of the 44 digits released in the data set, the most common digit was the ring finger (17), closely followed by the little finger (16), and then the middle finger (9). Only a single release was performed on the index finger and the thumb web space. Contractures affecting the metacarpal phalangeal joint were more numerous (37) than proximal interphalangeal joints (29).

A significant proportion of patients (19) in the study were retired or unemployed. Of the ten patients who worked, eight performed manual jobs. The time for return to work or activity with no restrictions ranged from 2 to 28 days post-procedure, with a median timeframe of 7 days. All patients reported either partial or complete improvement at six weeks and three months following PNA. With a median Southampton score of 2 out of 20 (range 0–14) and URAMs 2 out of 45 (range 0–29). At three months, PROMs demonstrated minimal discomfort and very limited or no impact of DD on function. There were no major complications or side effects to report. Nine minor skin tears, all less than four millimetres, healed with no infection and simple dressings. One case reported hypersensitivity in the follow-up period with distribution across unaffected digits and the unaffected hand; therefore, it was unlikely related to the procedure.

Baseline Tubiana grades for patients included fourteen-stage I (total passive extension deficit 0–45 degrees), eight-stage II (46–90 degrees), five-stage III (91–135 degrees), and two-stage IV (>135 degrees) [12]. Extension deficits at the MCPJ improved from an average deficit of 30 degrees to 4 degrees, and PIPJ from 37 degrees to 10 degrees over the three months (Table 2).

**Figure 1:**
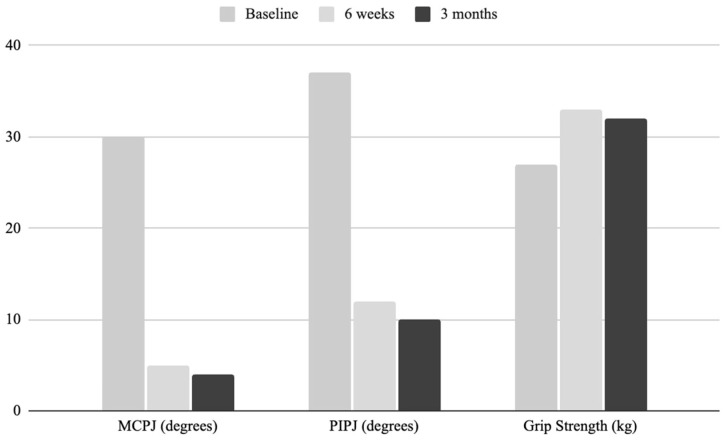
Extension deficit and grip strength changes with time.

The baseline average grip strength comparison for affected and unaffected hands was 28 and 29 kg, respectively (Table 2). Detailed individual patient data are available in Supplementary Table S1. Baseline contralateral grip strength was performed (*n* = 24). Fifty-eight per cent of patients were less than ten per cent stronger on the affected dominant hand. Sixteen of the twenty patients showed an improvement in their grip strength from baseline to three months (Figure 1). With an average overall improvement of 5.8 kg at the three-month follow-up (*p*-value = 0.07), the greatest average improvements were observed in the Tubiana stage two and three groups, at 9.15 kg and 7.35 kg, respectively. Although subgroup analysis by Tubiana stage was limited by sample size, the greatest mean grip strength improvement was observed in the Tubiana stage two and three cohorts.

## 4. Discussion

This study suggests that there is a baseline difference in grip strength of approximately one kilogram on the affected side, and that PNA leads to an improvement in grip strength at six weeks and three months post-procedure. Additionally, there was a significant improvement in contracture release, with excellent outcomes at both the MCP and PIP joints, showing an average improvement of 26 degrees and 27 degrees, respectively.

The short study duration of three months resulted in no recorded disease recurrence, a common sequelae and drawback of the PNA procedure compared to the open fasciectomy approach [13]. Bystrom et al. (2022) show a recurrence rate of 45 per cent at five-year follow-up [8]. Four patients underwent a PNA between two and five years prior and elected to undergo a repeat PNA on the same digit due to recurrence; these patients reported a functional benefit from the initial PNA. Eaton (2014) suggests that the anatomical pattern and the biological aggressiveness of an individual’s DDs contribute to recurrence, and the minimally invasive options continue to be in demand for patients [14]. In support of this, Van den Berge et al. (2023) demonstrate that second and third PNA procedures are equally effective in improving total passive extension deficit as the initial intervention [13].

This group of Dupuytren’s patients exhibited known risk factors: half of the patients were active smokers, one-third of the patients reported drinking more than 10 standard drinks per week, and a quarter of the patients were diabetic, which was consistent with the literature [15,16,17]. A positive family history of DD [18,19,20] was reported in a quarter of the cases; most of these patients were younger at the time of first presentation [21,22]. Fifteen patients were Australian-born with Australian parents, and the remaining patients were born in or were descendants of the United Kingdom and Europe, including Austria, Greece, Sweden, the Netherlands, Romania, Poland, and Italy, where there are established genetic links and a higher prevalence of DD [20].

Compared to Dupuytren’s open fasciectomy approach, one of the key benefits of PNA is the expedited recovery, with reduced time off work and hobbies. Blake et al. (2021) report a median return-to-work timeframe following Dupuytren’s open fasciectomy of two weeks [23]. Meanwhile, of the ten patients still working, including eight who perform manual jobs, six underwent PNA on a Wednesday and returned to work on complete duties before the following Wednesday. This highlights how minimally invasive the PNA technique is and how it may enable the younger working population to access treatment that improves their hand function in a more timely fashion. Likewise, it allows patients who are older and have comorbidities access to treatment in an outpatient setting, enabling them to return to hobbies and regain hand function required for independence.

A low complication rate was observed with nine minor skin tears, reinforcing previous literature on the acceptable safety profile of PNA [2,5,6,7,9,11,24,25,26]. Furthermore, a study by Winberg and Turesson (2023) indicated that patients strongly preferred minimally invasive options over traditional open fasciectomy procedures [27]. Three patients elected to undergo PNA on their contralateral hand following their three-month follow-up, highlighting the positive outcomes in contracture improvement and overall hand function.

Overall grip strength is an important component of hand function and measures improvement following hand surgery or intervention. Several studies have documented improvements in grip strength, indicating enhanced hand function, following procedures such as post-carpal tunnel release, cubital tunnel release [28], and trigger finger release [29]. Two DD studies have documented baseline measurements for grip strength; however, they did not report follow-up data [25,26]. Despite its contribution to hand function, a grip strength review has not been explicitly targeted before and after DD PNA procedures. 

Baseline grip strength measures show a difference in grip strength of at least one kilogram compared to the unaffected hand. Furthermore, the reduction in baseline grip strength of the subjects was reflected in the right affected hand being less than 10 per cent stronger than the left unaffected hand [30]. In one case, the affected right hand did not register on the Jamar Dynamometer, compared with 8 kilograms on the left side, which at six-week follow up improved to 11 kg. Overall, the average grip strength of the cohort improved from 27 kg to 32 kg from baseline to three months. The grip strength demonstrated a positive trend, though it was not statistically significant (*p* = 0.07). The ulnar digits are known to play a significant role in grip strength. Methot, Cinchalkar, and Richards (2010) demonstrated that when the ring and little finger are physically excluded from grip, the mean grip strength is reduced by 55 percent [31]. The grip strength of the PNA group that involved the ring finger and little finger improved by 10.5 and 4.8 kg, respectively. Alternatively, MacDermid et al. (2004) postulate that the middle finger contributes most significantly to grip strength, as measured using a specialised dynamometer that loads individual digits [32]. Of the nine PNAs that involved the middle finger, four failed to attend final follow-up, and there was an average reduction of 0.6 kg of grip strength at three months in the remaining five cases. Note, this study did not measure flexion deficits and the ability to make a full composite fist. Stiffness and reduced flexion may have contributed to reduced grip strength post-procedure, especially during the standard three-month period when a night splint was worn [10]. Only four cases in this series recorded reduced grip strength. These DD cases were considered more complex, two undergoing multi-digit release, and two with severe PIPJ contractures of 80 to 90 degrees before intervention. Despite the decreased objective measures, these patients reported functional improvement on PROMs at three-month follow-up.

This study’s limitations include its small sample size and short-term follow-up period. A larger cohort study with longer longitudinal data would establish whether the improvement in grip strength is consistent for most patients following PNA and confirm whether any consistent patterns emerge with specific digit involvement. Long-term follow-ups of 24 months or more would help establish whether joint contractures recur and whether grip strength gains are sustained. Furthermore, the inclusion of patients undergoing repeat procedures introduces heterogeneity, and separate analysis was not performed due to the small sample size. Another limitation of this study is the absence of a comparison group, such as patients undergoing open fasciectomy or collagenase injection, or untreated individuals. Without a control arm, we cannot definitively attribute grip strength changes to the procedure. Future studies incorporating a comparative design are recommended to strengthen causal inference. Another notable limitation of this study is the inclusion of patients with partial follow-up data, which deviated from our initially stated criterion of a minimum three-month follow-up. This was conducted to avoid unnecessary exclusion of patients with valid data at earlier timepoints and to reflect real-world follow-up patterns. However, all analyses involving three-month outcomes were strictly limited to patients with complete data at that timepoint to maintain analytical accuracy.

## 5. Conclusions

This study highlights the potential of percutaneous needle aponeurotomy as an effective and minimally invasive treatment for Dupuytren’s disease. Patients demonstrated improvements in grip strength and joint extension deficits, along with a rapid recovery, returning to daily activities within a median of seven days. These early findings suggest a functional benefit from the procedure; however, as the results did not reach statistical significance, they should be interpreted as indicative rather than definitive. The low complication rate and observed trends toward enhanced grip strength at three months reinforce the promise of this technique. Larger, adequately powered studies with extended follow-up are needed to confirm the durability and long-term benefits of percutaneous needle aponeurotomy. As the population continues to age and physical demands increase among the workforce, minimally invasive treatments such as this will play an increasingly valuable role in restoring hand function and improving quality of life.

## Figures and Tables

**Table 1:** Baseline characteristics of study participants.

Patients, *n* = 29	
Age (years), Median (range)	64 (46–92)
Gender, *n*	
Male	24
Female	5
Medical comorbidities, *n*	
Type 2 Diabetes	7
Heart disease	4
Social history, *n*	
Smoker	14
Excess Alcohol > 10 drinks pw	10
Working (manual:non-manual)	10 (8:2)
Family history, *n*	7
Affected hand, *n*	
Left	8
Right	21
Dominant hand	21
Digits involved, *n*	
Thumb	1
Index	1
Middle	9
Ring	17
Little	16
Affected joints, *n*	
MCPJ	37
PIPJ	29

Abbreviations: MCPJ = metacarpal phalangeal joint, PIPJ = proximal interphalangeal joint, pw = per week.

**Table 2:** Joint Contracture (Extension Deficit in Degrees) and Grip Strength (kgf) at Baseline, 6 Weeks, and 3 Months Post-PNA. Grip strength was measured using a Jamar Dynamometer. Negative extension values were corrected to zero. Data presented as mean, range (median). *n* = 20 at 3 months due to loss to follow-up.

Outcome Measure	Baseline	6 Weeks Post-PNA	3 Months Post-PNA	*p*-Value (vs. Baseline)
Metacarpophalangeal Joint	30° (0–90°; 20°), *n* = 37	5° (0–30°; 0°), *n* = 30	4° (0–40°; 0°), *n* = 25	–
Proximal Interphalangeal Joint	37° (0–90°; 30°), *n* = 29	12° (0–60°; 10°), *n* = 28	10° (0–40°; 0°), *n* = 19	–
Grip Strength (kgf)	27 (0–50; 28), *n* = 29	33 (11–52; 32), *n* = 26	32 (13–57; 32), *n* = 20	0.09 (6 weeks), 0.07 (3 months)

## Data Availability

The data presented in this study are available on request from the corresponding author due to privacy restrictions.

## Supplementary Materials

The following supporting information can be downloaded at: https://www.mdpi.com/article/10.3390/jcm14124171/s1, Table S1: Full data set including grip strength and joint range of motion measures at baseline, six weeks and three months.

## Abbreviations

The following abbreviations are used in this manuscript:

DD	Dupuytren’s Disease
PNA	Percutaneous Needle Aponeurotomy
MCPJ	Metacarpophalangeal Joint
PIPJ	Proximal Interphalangeal Joint
PROMs	Patient-Reported Outcome Measures
URAM	Southampton Unité Rhumatologique des Affections de la Main
kgf	Kilograms-force
pw	Per Week
ROM	Range of Motion
