# Supplementary material for: Dupuytren’s Disease Percutaneous Needle Aponeurotomy: Does Grip Strength Improve Post Procedure?
Source: J Clin Med. 2025 Jun 12;14(12):4171. doi: 10.3390/jcm14124171 (PMC12194114; doi:10.3390/jcm14124171)
Supplement: Supplementary file 1 [file jcm-14-04171-s001.zip › jcm-3646881-supplementary.pdf]

Journal of
Clinical Medicine

MDPI

Table S1. Full data set including grip strength and joint range of motion measures at baseline, six weeks and three months.

Hand	Finger(s)	MCP	PIPJ	DIPJ	Tubiana grades	affected GS	unaffected GS	Affected GS	Change in GS	MCP	PIP	DIP	Affected GS	Change in GS from w0	MCP	PIP	DIP
Left	LF	10	45	0	2	**37.4**	-	**46.7**	**9.3**	0	20	0			0	25	0
Left	thumb	15	15		1	**12.8**	-	**13.2**	**0.4**	0	0		**13.2**	**0.4**	0	0	
Right	LF	0	45	0	2	**31.8**	-	**36.2**	**4.4**	0	20	0	**37.2**	**5.4**	0	30	0
Left	RF	40	20	-10	2	**12.6**	-	**30.1**	**17.5**	5	0	0	**30.4**	**17.8**	20	0	0
Right	RF	45	90	0	3	**1.3**	-	**25.4**	**24.1**	0	45	0	**25.1**	**23.8**	0	30	0
Right	MF	30	10	0	1	**34**	27	**28.8**	**-5.2**	30	0	0					
	RF	20	20	0						0	0	0					
Right	LF	0	90	0	3	**33**	31	**38.2**	**5.2**	0	30	0	**31.2**	**-1.8**	0	40	0
Right	LF	30	10	0	1	**29**	31	**30.8**	**1.8**	0	10	0	**35**	**6**	5	0	0
Right	IF	35	10	40	2	**41**	38	**35**	**-6**	0	20	0	**46**	**5**	0	20	0
Right	RF	20	20	0	1	**50**	54	**49**	**-1**	10	0	0	**57**	**7**	0	0	0
Right	MF	30	0	0	1	**15.4**	16.4						**11.6**	**-3.8**	5	0	0
	RF	20	0	0											0	0	0
Right	RF	0	30	0	1	**27.3**	43.9	**45.7**	**18.4**	0	5	0	**40**	**12.7**	0	0	0
Right	MF	50	80	10	4	**46.7**	38.7	**45.1**	**-1.6**	0	15	0	**45.1**	**-1.6**	0	15	0
Right	LF	20	80	30	3	**15.4**	11.7	**15.2**	**-0.2**	0	20	0					
Left	LF	60	20	-10	2	**29.2**	27.1	**28.9**	**-0.3**	10	0	0					
Left	RF	20	40	0	2	**16.8**	21						**24**	**7.2**	5	0	0
	LF	10	0	0											0	0	0
Right	RF	30	0	0	1	**48.5**	38.5	**51.7**	**3.2**	0	0	0			0	0	0
Left	LF	20	40	0	2	**27.8**	20.7	**37.2**	**9.4**	0	10	0	**40**	**12.2**	0	0	0
Right	RF	20	0	0		**19**	15.1	**27.7**	**8.7**	10	0	0	**26.3**	**7.3**	0	0	0
	LF	30	50	0	2					20	10	0			10	15	0

Right	MF	0	10	0	1	**25.4**	21.4	**29.2**	**3.8**	0	0	0	**32**	**6.6**	0	0	0
	RF	10	10	0						0	10	0			0	10	0
	LF	20	20	0						0	0	0			0	0	0
Right	LF	30	0	0	1	**42**	41	**51**	**9**	0	0	0	**44**	**2**	0	0	0
Right	MF	10	0	0	1	**20.9**	28	**21.6**	**0.7**	0	0	0	**14.4**	**-6.5**	0	0	0
	RF	10	0	0						5	0	0			10	10	0
Right	MF	30	0	0	1	**35**	36.3	**40.8**	**5.8**	10	0	0					
	RF	30	0	0						10	0	0					
	LF	30	90	0						20	10	0					
Right	LF	30	30	0	3	**37**	31.1	**35.3**	**-1.7**	5	10	0	**42**	**5**	5	0	0
Right	MF	80	60	0	4	**0**	8	**10.9**	**10.9**	0	40	0					
	RF	80	60	0						20	60	0					
Right	MF	30	10	0	1	**21.2**	41.7	**32.9**	**11.7**	0	0	0					
	RF	30	10	0						0	0	0					
	LF	20	0	0	1					0	5	0					
Left	RF	0	40	0	1	**31.5**	34.6	**26.3**	**-5.2**	0	5	5					
Left	MF	5	0	0		**17.6**	21						**20**	**2.4**	0	0	0
	RF	20	0	0											0	0	0
	LF	90	0	0	3										40	20	0
Right	RF	0	0	0		**19.2**	22	**22.4**	**3.2**	0	0	0	**28**	**8.8**	0	0	0
	LF	0	10	0	1					0	0	0			0	0	0

*IF = index finger, MF = middle finger, RF = ring finger, LF = little finger.
